# Treatment of venous thromboembolism – effects of different therapeutic strategies on bleeding and recurrence rates and considerations for future anticoagulant management

**DOI:** 10.1186/1477-9560-10-24

**Published:** 2012-12-31

**Authors:** Bastian Hass, Jayne Pooley, Adrian E Harrington, Andreas Clemens, Martin Feuring

**Affiliations:** 1 Boehringer Ingelheim GmbH, Binger Strasse 173, 55216, Ingelheim am Rhein, Germany; 2 United BioSource Corporation, River House, 33 Point Pleasant, London, SW18 1NN, UK; 3 Boehringer Ingelheim Pharma GmbH & Co. KG, Binger Strasse 173, 55216, Ingelheim am Rhein, Germany; 4 Center of Thrombosis and Hemostasis, Johannes Gutenberg University, Medical Center, Mainz, Germany

**Keywords:** Venous thromboembolism, Anticoagulants, Vitamin K antagonists, Heparin, Recurrence, Bleeding

## Abstract

Effective treatment of venous thromboembolism (VTE) strikes a balance between prevention of recurrence and bleeding complications. The current standard of care is heparin followed by a vitamin K antagonist such as warfarin. However, this option is not without its limitations, as the anticoagulant effect of warfarin is associated with high inter- and intra-patient variability and patients must be regularly monitored to ensure that anticoagulation is within the narrow target therapeutic range. Several novel oral anticoagulant agents are in the advanced stages of development for VTE treatment, some of which are given after an initial period of heparin treatment, in line with current practice, while others switch from high to low doses after the initial phase of treatment. In this review we assess the critical considerations for treating VTE in light of emerging clinical data for new oral agents and discuss the merits of novel treatment regimens for patients who have experienced an episode of deep vein thrombosis or pulmonary embolism.

## Review

Vitamin K antagonists (VKAs) such as warfarin have been the mainstay of treatment and secondary prevention of venous thromboembolism (VTE) for many years, and are recognised by international guidelines as the current standard of care. VKAs usually establish an anticoagulant effect within 2–3 days of administration. However, because a rapid, intensive anticoagulant effect is required, a quicker acting agent such as heparin is used initially until the desired anticoagulant effect of the VKA has been achieved. Guidelines on the management of VTE recommend that administration of heparin is started concomitantly with a VKA and discontinued after 5 days or more, once the international normalised ratio (INR) has been between 2.0 and 3.0 for 2 consecutive days [1].

Despite the benefits offered by oral therapy, the anticoagulant effect of VKA treatment is associated with significant inter- and intra-patient variability, leading to unpredictable results in clinical practice. In addition, VKAs have an unpredictable dose–response relationship. Furthermore, regular intensive blood monitoring is required to ensure that the INR is maintained within the target therapeutic range (INR 2.0-3.0); under-anticoagulation can result in recurrent thromboembolism, while over-anticoagulation increases the risk of bleeding. Achieving a balance between the risk of recurrence and bleeding complications is therefore a central consideration in VTE management.

Several novel, oral anticoagulants are in development, including dabigatran etexilate (dabigatran; a reversible direct thrombin inhibitor) and the factor Xa inhibitors apixaban, edoxaban and rivaroxaban. These anticoagulants could provide a more predictable alternative to VKAs and have the potential to change the recommended standard for treatment of VTE.

### What is known about the rate of VTE recurrence in patients treated with the currently-recommended therapeutic agents?

**Figure 1:**
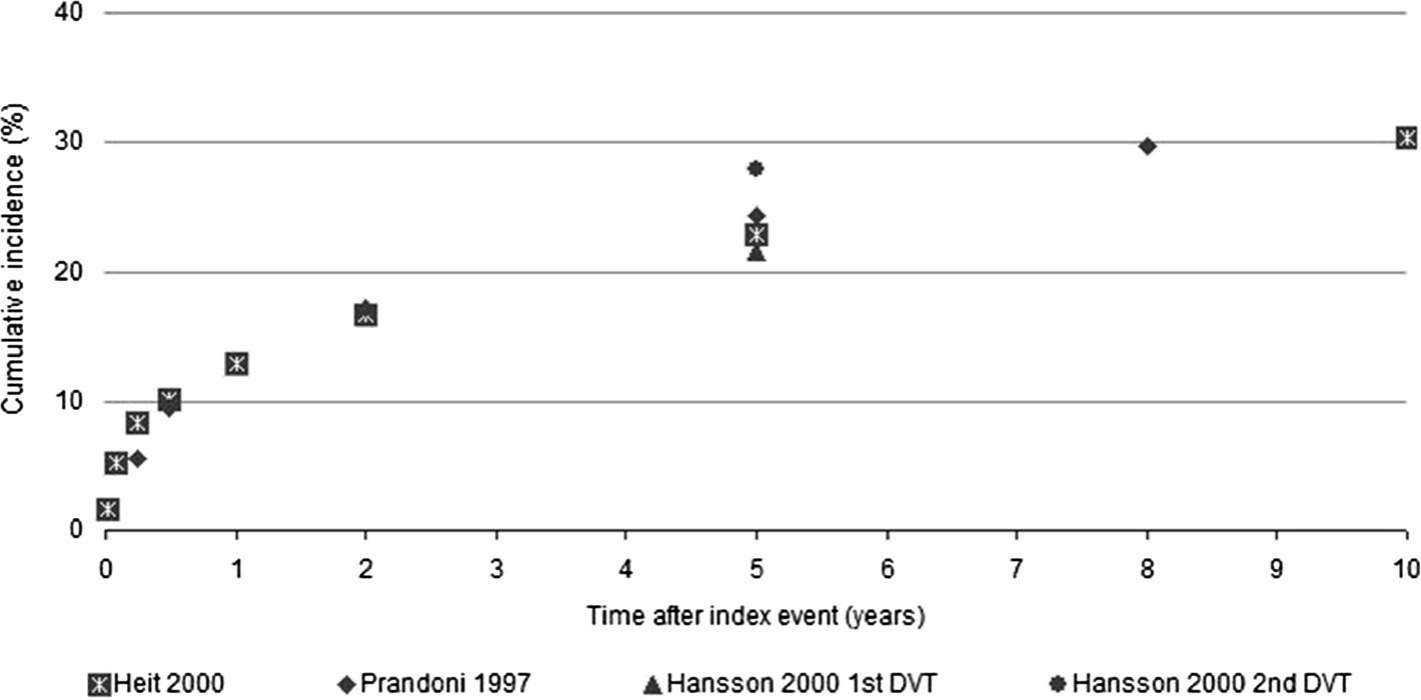
**Rates of VTE recurrence in registry patients**[3-5].

Data on the frequency of early recurrence of VTE (i.e., within 5 days of treatment initiation) are sparse and associated with broad confidence intervals [2]. However, clinical trials and patient registries have consistently demonstrated that the rate of VTE recurrence is highest immediately after the initial event and gradually decreases over time (Figure 1) [3-5]. In one analysis of recurrent VTE timing among 1021 patients with deep vein thrombosis (DVT) or pulmonary embolism (PE) who received heparin plus warfarin, there was a clustering of episodes within the first 2–3 weeks after treatment initiation [6]: 

26% occurred within 7 days (cumulative incidence 1.5%)

57% within 14 days (cumulative incidence 3.2%)

72% within 21 days (cumulative incidence 4.1%)

Cumulative incidence of recurrence in these patients reached a plateau of 6% at 3 months [6].

In a meta-analysis of 18 studies that addressed the timing of recurrent VTE in patients who were continuously treated with VKAs for 1–6 months after their first thromboembolic event, the incidence of recurrence stabilised approximately 9 months after the index event and appeared to be independent of the duration of anticoagulant therapy [7].

The optimal duration of anticoagulant therapy is controversial. According to the American College of Chest Physicians (ACCP) guidelines, the risk of recurrence after stopping therapy is largely determined by two factors; whether the acute episode of VTE has been effectively treated and the patient’s intrinsic risk of having a new episode [1]. Patients with reversible provoking risk factors for VTE (major factors such as surgery, hospitalisation or plaster cast immobilisation, or minor factors such as oestrogen therapy, pregnancy or prolonged travel, prior to the acute episode) clearly benefit from anticoagulation for 3 months rather than shorter durations. The guidelines identify unprovoked VTE and active cancer as the most important risk factors for recurrence after stopping anticoagulant therapy and patients in these categories should generally receive longer durations of thromboprophylaxis. It is recommended that the risk-to-benefit ratio of continuing long-term anticoagulant therapy is assessed periodically in the individual patient [1].

The pattern of recurrence following a first unprovoked VTE is different in men and women. In a meta-analysis of prospective studies of patients with a first VTE who received standardised anticoagulant treatment, among the patients with an unprovoked VTE, men were at significantly higher risk of recurrence than women (hazard ratio [HR] 2.2, 95% confidence interval [CI] 1.7, 2.8) [8].

It appears that the risk of developing recurrent VTE is not related to the type of initial thromboembolic event (i.e., DVT or PE). A systematic review showed that the overall risk of a recurrent thromboembolic event was not significantly different between patients presenting with PE or DVT. It is therefore unlikely that the distribution of recurrent events would be different between these two groups of patients [9]. Analysis of patients in the Worcester Venous Thromboembolism Study found that those who presented with PE or isolated DVT experienced similar rates of subsequent PE and overall VTE in the 3-year follow-up (5.9% vs. 5.1% and 15.0% vs. 17.9%, respectively) [10]. However, the risk of early mortality from VTE (i.e., within 1 month) is much greater after presenting with PE than DVT [11]. Furthermore, recurrent episodes of VTE are approximately three times more likely to be PE after an initial PE (~60%) than after an initial DVT (~20%) [11,12]. This difference may justify more aggressive initial treatment for patients presenting with PE [1].

### How do bleeding rates vary in patients treated with currently recommended therapeutic agents?

Bleeding is the primary complication of anticoagulant therapy. In the earlier (placebo-controlled) trials in patients with VTE or atrial fibrillation, major bleeding occurred in approximately 1–1.5% of patients per year during 3–12 months of VKA therapy compared with 0.6-1% for controls. Rates of intracranial haemorrhage (ICH) were 0.3% and 0.1%, respectively. Thus, it is estimated that treatment with VKAs increases the risk of major bleeding by 0.3-0.5% per year and of ICH by approximately 0.2% per year compared with controls [13-15]. However, higher major bleeding rates of 2-4% per year have been reported during longer-term trials of VKAs in patients with VTE [15-17]. In the VKA treatment arms of recent clinical trials of VTE treatment or secondary prevention, approximately of 2% of patients experience major bleeding [18-22]. Recent trials in patients with atrial fibrillation show rates of ICH of 0.7-0.8% per year with VKA therapy [23-25].

The choice of heparin formulation, i.e., subcutaneous weight-adjusted unfractionated heparin (UFH) or low-molecular-weight heparin (LMWH), has no observed effect on the frequency of major bleeding [26,27]. Several studies of VTE-treated patients have also shown a clustering of bleeding events at the start (i.e., during the initial 3 months) of anticoagulant therapy [6,28-30]. This is consistent with the hypothesis that patients with a predisposition to bleeding are more likely to develop this complication soon after anticoagulant initiation [30].

Due to their selective nature, clinical trials may, however, underestimate the frequency of bleeding, and the incidence of bleeding is markedly greater in clinical practice. The Worcester VTE study, a large US community-based trial, showed major bleeding rates of 12-13% per year, and the cumulative incidence of major bleeding was approximately 8% in the month following initial diagnosis of VTE [31].

### What are the consequences of anticoagulant-related bleeding?

The clinical impact of anticoagulant-related bleeding complications is often severe; as many as 11% of major bleeding episodes are fatal within 3 months [32]. Among 17,368 patients with VTE enrolled on the Registry of Patients with Venous Thromboembolism (RIETE), 2.3% developed major bleeding during the first 3 months of therapy [33]. Of those patients with bleeding episodes, 5.9% re-bled within 30 days, all at the same site as the first episode. Furthermore, 33% died within 30 days of major bleeding, with the majority of deaths due to bleeding [33]. In a meta-analysis of trials with more than 6 months’ follow-up, the case-fatality rate for major bleeding was 13.4%; for intracranial bleeding, 45.8% of episodes were fatal [30].

Bleeding episodes during anticoagulant therapy are also associated with decreased quality of life and increased resource utilisation and costs. In a randomised controlled trial of warfarin for the prevention of stroke in non-rheumatic atrial fibrillation, patients taking warfarin who had a bleeding episode had significantly lower scores on a summary health perceptions index (p < 0.05), and significantly higher scores for health concern and health distress (p < 0.05) compared with patients who did not bleed [34]. Furthermore, in a model of the costs of acute DVT treatment, minor bleeding complications were assumed to result in 1 additional day of hospitalisation and physician services for subsequent hospital care, at a cost of $499 [35].

### What factors are associated with unfavourable outcomes among patients with VTE?

Many studies have investigated the factors associated with an increased risk of VTE recurrence. The most strongly implicated are active cancer, increased plasma fibrin D-dimer, male sex, increasing patient age, increasing body mass index, lupus anticoagulant, antiphospholipid antibody, and protein C or protein S deficiency [4,16,36-41]. Moreover, patients who experience a recurrence of VTE during VKA therapy have a worse prognosis (i.e., increased incidence of further recurrences and bleeding) than those with new VTE or those who suffered recurrence after termination of VKA therapy.

The major determinants of VKA-induced bleeding are the intensity of the anticoagulant effect, patient characteristics, concomitant use of drugs that interfere with haemostasis and length of therapy [15]. In a multivariate analysis of the RIETE registry, age >75 years, recent bleeding, cancer, creatinine levels >1.2 mg/dL, anaemia, or PE at baseline were independently associated with an increased risk for major bleeding, but type or number of concomitant antithrombotic agents received were not [42]. When risk profiles were derived by assigning scores to these factors, the incidences of major bleeding in the first 3 months of therapy were 0.3%, 2.6% and 7.3%, in low-, medium- and high-risk patients, respectively [42].

In patients receiving anticoagulant therapy, those with cancer have an increased risk of bleeding complications compared with patients without cancer [43-46]. In one study that evaluated outcomes in 842 patients with VTE who were treated with heparin and VKAs, 181 of whom had cancer, the 12-month cumulative incidence of major bleeding in patients with cancer was more than twice that of patients without cancer (HR, 2.2; 95% CI 1.2, 4.1). Moreover, the frequency of major bleeding correlated with the severity of the malignancy [45]. The incidence of bleeding also varies according to the site of cancer [45,46], with one study showing that genitourinary cancers were most strongly associated with major bleeding (HR, 4.5; 95% CI 2.1, 9.9) [45].

In patients with impaired renal function, LMWH (which is mainly cleared by the kidneys) may bioaccumulate and cause bleeding, although the evidence for excluding patients with renal insufficiency is controversial. Moreover, it has been reported that the risk of major bleeding in patients with impaired renal function is increased with both LMWH and UFH [47].

### How do anticoagulant strategies differ in high-risk patient populations?

Patients with cancer represent a population at particularly high risk for VTE. Cushman et al. found the first-year incidence of recurrent VTE in the general VTE population was 7.7%, compared with 14.0% in patients with cancer [37]. Among patients enrolled in the RIETE registry, those with cancer had an increased incidence of recurrent VTE (11.4% vs 2.1%; p < 0.001), major bleeding (5.1% vs. 2.1%; p = 0.007) and mortality (20% vs 5.4%; p < 0.001), compared with patients without cancer [48].

Management of cancer patients with VKAs can be challenging because of the frequent use of chemotherapy and other drugs that may affect INR control and increase bleeding risk through induced thrombocytopenia, in addition to increasing the risk of liver dysfunction [49]. To determine whether outcomes in VTE patients with cancer were better with LMWH monotherapy or VKAs, a meta-analysis of five open-label secondary prevention trials was performed (two each of 3 and 6 months’ duration, one unspecified). The pooled risk ratio for VTE recurrence in patients treated with LMWH was 0.53 (95% CI 0.36, 0.76; p = 0.007) and the pooled relative risk for major bleeding was 0.98 (95% CI 0.49, 1.93; p = 0.95) [49]. The American Society of Clinical Oncology guidelines recommend the use of LMWH monotherapy in patients with cancer who experience VTE, for both initial (5–10 days) and long-term (≥6 months) treatment [50].

There is also clinical interest in an effective and predictable treatment regimen for patients with more severe VTE (e.g. acute PE), that removes the need for laboratory monitoring associated with VKAs and minimises bleeding complications. Mortality from recurrent VTE is 2- to 3-fold greater after PE than DVT [9,51]. In a study of patients with acute symptomatic PE (cancer patients excluded), the hospital length of stay was shorter in patients on LMWH monotherapy compared with those receiving warfarin, although for VTE recurrence, death, bleeding episodes or a composite of the three, there were no significant differences between treatment groups [52].

### How does treatment with novel oral anticoagulants deviate from the established treatment paradigm?

Novel oral anticoagulants including apixaban, dabigatran, edoxaban and rivaroxaban can be grouped into two categories depending on the VTE treatment regimens used in clinical trials.

In trials of dabigatran, patients began treatment with heparin in addition to warfarin (or warfarin-placebo) for at least 5 days, until the patient’s INR or sham INR reached therapeutic levels for 2 consecutive days, at which point dabigatran was initiated at a dose of 150 mg twice daily (bid) [21,22]. Similarly, ongoing studies of edoxaban begin oral administration of 60 mg once daily (qd) following initial heparin treatment once a stable therapeutic INR has been achieved with warfarin [53].

In the cases of rivaroxaban and apixaban, oral therapy is initiated without concomitant heparin. In the EINSTEIN trials, patients began treatment with 15 mg rivaroxaban bid, reduced to 20 mg qd after 3 weeks [18,19]. A similar strategy is used in the AMPLIFY trial, where the dose of apixaban is reduced from 10 mg bid to 5 mg bid after 7 days [54].

### What are the rates of bleeding and recurrence in patients treated with novel oral anticoagulants?

Data published to date suggest that initiation of treatment with heparin has a crucial role to play in minimising the early rate of VTE recurrence. In a study that compared treatment with the pentasaccharide idraparinux with standard therapy (i.e., heparin followed by VKA) in patients with acute PE, the rates of VTE recurrence during the first 2 weeks of treatment favoured standard therapy [55]. This effect was maintained throughout the study; the odds ratio for recurrence by day 92 was 2.14 (95% CI 1.21, 3.78), suggesting that initial heparin treatment may have a durable protective effect [55]. Results from recent trials of rivaroxaban, where initiation of treatment with heparin did not occur [18,19], are challenging this concept, though the evidence is still too scarce to be conclusive.

Recurrence rates reported in phase III clinical trials of novel oral anticoagulants for patients presenting with acute VTE are shown in Table 1. Overall, the incidence of VTE recurrence in these studies is low and not significantly different from that in the comparator LMWH plus VKA arms – typically 2-3% at 3 months. The cumulative risk of VTE recurrence in patients presenting with DVT and/or PE over time was similar for LMWH plus dabigatran and LMWH plus warfarin in the RE-COVER and RE-COVER II studies [21,22]. In the EINSTEIN studies, rivaroxaban was non-inferior to standard therapy in terms of cumulative recurrence of VTE in patients presenting with DVT and in patients presenting with PE [18,19]. No data on the recurrence rates in patients treated with edoxaban or apixaban are available, although studies are ongoing. 

**Table 1:** Rates of recurrent VTE and bleeding reported in clinical studies of novel oral anticoagulants

**Study**	**Patients, N**	**Treatment duration**	**Rate of VTE recurrence**	**Rate of bleeding**		
				***Major bleeding***	***All bleeding***	***Major/CRNM bleeding***
Schulman 2009 RE-COVER [21]	2564	6 months	Dabigatran 2.4% LMWH + VKA 2.1% **Difference 0.4% (95% CI −0.8%, 1.5%); p < 0.001, non-inferior***HR 1.10 (95% CI 0.65, 1.84)*	^***a ***^Dabigatran 1.6% LMWH + VKA 1.9% HR 0.82 (95% CI 0.45, 1.48)	Dabigatran 16.1%LMWH + VKA 21.9% HR 0.71 (95% CI 0.59, 0.85)	^***b ***^Dabigatran 5.6% LMWH + VKA 8.8% HR 0.63 (95% CI 0.47, 0.84); p = 0.002
Schulman 2011 RE-COVER II [22]	2568	6 months	Dabigatran 2.4% LMWH + VKA 2.2% **Difference 0.2% (95% CI −1.0%, 1.5%); p < 0.0001, non-inferior***HR 1.08 (95% CI 0.64, 1.80)*	^***a ***^Dabigatran 1.2% LMWH + VKA 1.7% HR 0.69 (95% CI 0.36, 1.32)	Dabigatran 15.6% LMWH + VKA 22.1% HR 0.67 (95% CI 0.56, 0.81)	
EINSTEIN Investigators 2010 EINSTEIN-DVT [18]	3451	3, 6 or 12 months	Rivaroxaban 2.1% LMWH + VKA 3.0% **p < 0.001, non-inferior***HR 0.68 (95% CI 0.44, 1.04)*	^***c ***^Rivaroxaban 0.8% LMWH + VKA 1.2% HR 0.65 (95% CI 0.33, 1.30); p = 0.21		^***d ***^Rivaroxaban 8.1% LMWH + VKA 8.1% HR 0.97 (95% CI 0.76, 1.22); p = 0.77
EINSTEIN-PE Investigators 2012 EINSTEIN-PE [19]	4832	3, 6 or 12 months	Rivaroxaban 2.1% LMWH + VKA 1.8% **p = 0.003, non-inferior***HR 1.12 (95% CI 0.75, 1.68)*	^***c ***^Rivaroxaban 1.1% LMWH + VKA 2.2% HR 0.49 (95% CI 0.31, 0.79); p = 0.003		^***d ***^Rivaroxaban 10.3% LMWH + VKA 11.4% HR 0.90 (95% CI 0.76, 1.07); p = 0.23

CRNM, clinically relevant non-major; HR, hazard ratio; LMWH, low molecular weight heparin; VKA, vitamin K antagonist.

^a^: Bleeding defined as major if clinically overt and if it was associated with a fall in the haemoglobin level of at least 20 g/L, resulted in the need for transfusion of 2 or more units of red cells, involved a critical site or was fatal.

^b^: Clinically relevant non-major bleeding was defined as bleeding not meeting the criteria for major bleeding but associated with spontaneous skin haematoma of at least 25 cm^2^, spontaneous nose bleed of more than 5 minutes’ duration, macroscopic haematuria (spontaneous or, if associated with intervention, lasting more than 24 hours), spontaneous rectal bleeding (more than spotting on toilet paper), gingival bleeding for more than 5 minutes, bleeding leading to hospitalisation and/or requiring surgical treatment, bleeding leading to a transfusion of less than 2 units of whole blood or red cells, or any other bleeding considered clinically relevant by the investigator.

^c^: Bleeding was defined as major if it was clinically overt and associated with a fall in the haemoglobin level of 20 g/L or more, or if it led to transfusion of two or more units of red cells, or if it was retroperitoneal, intracranial, occurred in a critical site or contributed to death.

^d:^ Clinically relevant non-major bleeding was defined as overt bleeding not meeting the criteria for major bleeding but associated with medical intervention, unscheduled contact with a physician, interruption or discontinuation of study treatment, or associated with any other discomfort such as pain or impairment of activities of daily life.

According to a meta-analysis of selected trials of dabigatran etexilate, there was a higher incidence of acute coronary syndrome (ACS) or myocardial infarction (MI) events in the dabigatran arms compared with the control arms; however, the trials included several doses, comparators, indications and treatment durations [56]. These findings were mainly driven by the large RE-LY trial in patients with atrial fibrillation in which there were numerically, but not statistically significantly, fewer MIs with warfarin compared with dabigatran. ACS events were also more frequent (but still < 1%) with dabigatran than with warfarin in a trial of secondary prevention of VTE, whereas there was no difference between dabigatran and placebo during extended maintenance therapy after VTE [57,58]. In addition, no signal for increased ACS events was detected with dabigatran versus enoxaparin in pooled trials of primary VTE prevention in patients after total hip or knee replacement [59]. This suggests that the difference seen in the warfarin-controlled studies may be due to a more protective effect of warfarin rather than an adverse effect of dabigatran. In the VTE treatment trials, the incidences of ACS events in the dabigatran and standard therapy groups, respectively, were 0.3% versus 0.2% in RE-COVER and 0.4% versus 0.1% in RE-COVER II [21,22]. In the EINSTEIN-PE study, incidences of ACS were 0.6% with rivaroxaban versus 0.9% with standard therapy [19].

Reported rates of bleeding complications in clinical trials for the novel oral agents in development are also shown in Table 1. Dabigatran was associated with similar major bleeds, and significantly fewer episodes of any bleeding compared with warfarin in the RE-COVER study [21]. A similar pattern was observed in the RE-COVER II study [22]. These findings are consistent with previous data from phase III studies of dabigatran, such as the RE-LY trial [23]. In the EINSTEIN-DVT trial, there was no significant difference between rivaroxaban and warfarin regarding the rate of the primary safety endpoint (the composite of major or clinically relevant non-major bleeding), or in the incidence of major bleeding [18]. In EINSTEIN-PE, fewer major bleeding events were observed with rivaroxaban than with standard therapy, although there was no significant difference between treatment groups in terms of the composite of major or clinically relevant non-major bleeding [19]. Without a direct comparative trial of the new oral anticoagulants with consistent definitions of bleeding and VTE recurrence and independent assessments of all endpoints it is not valuable, and would have to be interpreted with significant caution, when attempting to determine whether one drug is more effective or safe than another.

### What does the available evidence mean for the future treatment of VTE?

To date, initial heparin followed by VKA has shown the greatest benefit in reducing the rate of recurrent VTE. This regimen therefore represents the current gold standard against which any new treatment will be assessed. However, several studies have shown that many patients experience under- or over-anticoagulation while on VKA therapy, putting them at risk of recurrent VTE or bleeding, respectively. A recent study of discharge records for patients in the United States hospitalised with an index VTE found that 19.8% had no INR monitoring and for those who were monitored only 38.1% of INR values were within the therapeutic range (INR 2.0-3.0) [60]. In recent clinical trials, the time patients receiving warfarin spent in the therapeutic range is 58% in EINSTEIN-DVT and 60% in RE-COVER and the level of anticoagulation in these patients at any given point in the therapy is subject to uncertainty.

There is a lack of information from patients receiving anticoagulation for VTE treatment in real-world settings. Therefore we provide some information from other patient groups with the caveat that they do not translate directly to patients with VTE. Several publications indicate that, although good levels of anticoagulation control can be achieved in routine practice [61,62], this is not universal. For example, time in therapeutic range among atrial fibrillation patients was 42.1% in ‘usual care’ (outside of specialist anticoagulation clinics) [63], and was 48.7% during the first 3 months of therapy after heart valve surgery in a retrospective cohort prior to implementing a trial of an ‘aggressive warfarin dosing algorithm’ [64]. Further to this, registry studies of VKA use demonstrate that only about 50% of atrial fibrillation patients at moderate or high risk of stroke actually receive treatment with a VKA. Reasons cited by physicians for not prescribing warfarin included previous haemorrhage (including ICH) while taking warfarin, falls and patient refusal or history of non-adherence [65,66].

Consideration of clinical trial data on heparin plus VKA should also take into account the likelihood that anticoagulation use and control may be poorer in real-world settings. Simply put, it is probable that event rates reported in the heparin plus VKA arms of clinical trials overestimate the potential performance in clinical practice. Additionally, the population of patients prescribed VKAs will be less homogeneous than the carefully selected clinical trial population, including a proportion who will be VKA-naïve, and thus less likely to experience good control after starting VKAs in clinical practice. While some of the factors potentially contributing to poorer anticoagulation usage, adherence and control in clinical practice compared with clinical trials may apply equally to the novel oral anticoagulants and to conventional therapy, others might be expected to apply more strongly to the outcomes of conventional therapy, namely those factors deriving from the complexity of optimally managing VKA therapy. In this context, demonstration of non-inferiority versus heparin plus VKA in studies of novel agents could be indicative of potentially improved outcomes in patients treated with these drugs compared with warfarin in clinical practice. However, there are currently no data available to evaluate this.

Dabigatran etexilate has been available for clinical use for longer than rivaroxaban. As expected with a new anticoagulant, several case reports of serious bleeding in patients with atrial fibrillation who were taking dabigatran have been published. However, a recent review of post-approval safety data by the European Medicines Agency found that the frequency of reported fatal bleedings with dabigatran was significantly lower than had been observed in clinical trials at the time of authorisation [67]. A US Food and Drug Administration safety analysis concluded that dabigatran, used in patients with atrial fibrillation, did not increase the risk of bleeding compared to warfarin, and found that the combined incidence of ICH and gastrointestinal bleeding for new users of warfarin was higher than for new dabigatran users [68]. In an observational real-world study, the efficacy and safety data for dabigatran in patients who had undergone total hip or knee replacement were also supportive of the phase III trial results [69].

Bleeding complications represent the primary safety concern for physicians treating patients with VTE. To date, data are failing to identify significant differences in bleeding rates in subgroups of patients who may respond better to alternative treatment strategies. Data are also scarce for patients at the extreme end of the spectrum with the most severe VTE (i.e., PE). Moreover, there remains a need to develop an effective method of linking patient bleeding risk profiles with a range of therapeutic options. Additional study data will help to differentiate the risks and benefits of the different anticoagulants and might enable identification of VTE patient subgroups that could benefit from alternative treatments.

The risks of VTE recurrence and bleeding are highest in the first weeks of treatment, which underscores the need to achieve the optimal level of anticoagulation in this crucial phase. Regardless of the treatment strategy, physicians must aim to achieve an optimal balance between efficacy and safety. A novel anticoagulant that demonstrates comparable efficacy in terms of VTE prevention compared with the standard of care, but may also minimise bleeding complications, has the potential to change current medical practice for the treatment of VTE.

## Conclusions

Novel oral anticoagulants have demonstrated safety and efficacy in clinical trials and could provide physicians and patients with DVT or PE more convenient therapeutic options. In clinical trials, on the key clinical outcomes of VTE recurrence and bleeding, these new agents perform similarly to the established standard of care, i.e., heparin plus VKA. However, in real-world settings, novel agents could produce better patient outcomes.

Some novel agents, such as rivaroxaban and apixaban, are initiated at higher doses without heparin, but clinical trial data have yet to confirm which patient populations are likely to derive more or less benefit from this alternative approach, especially in the first 2 weeks of treatment. The established treatment regimen – initial use of heparin followed by an oral anticoagulant – may well remain the standard of care in certain high-risk populations. However, it remains to be seen which approach will lead to the greatest improvements in clinical outcomes in the treatment and secondary prevention of VTE.

## Abbreviations

ACCP: American College of Chest Physicians; ACS: Acute coronary syndrome; Bid: Twice daily; CI: Confidence interval; CRNM: Clinically relevant non-major; DVT: Deep vein thrombosis; HR: Hazard ratio; ICH: Intracranial haemorrhage; INR: International normalised ratio; LMWH: Low-molecular-weight heparin; MI: Myocardial infarction; PE: Pulmonary embolism; QD: Once daily; UFH: Unfractionated heparin; VKA: Vitamin K antagonist; VTE: Venous thromboembolism.

## Competing interests

BH, AC and MF are employees of Boehringer Ingelheim. JP and AEH are employees of United BioSource Corporation. The authors declare that they have no competing interests.

## Authors’ contributions

BH conceived the review, contributed to planning and drafting, and reviewed the manuscript. JP drafted, edited and reviewed the manuscript. AEH carried out literature searches, drafted and edited the manuscript. AC contributed editorial direction and reviewed the manuscript. MF reviewed the clinical data presented in the manuscript and contributed editorial guidance. All authors read and approved the final manuscript.
